# Recent Developments in Nanoporous Graphene Membranes for Organic Solvent Nanofiltration: A Short Review

**DOI:** 10.3390/membranes11100793

**Published:** 2021-10-18

**Authors:** Yoon-Tae Nam, Jun-Hyeok Kang, Jae-Dong Jang, Jun-Hyuk Bae, Hee-Tae Jung, Dae-Woo Kim

**Affiliations:** 1 Department of Chemical and Biomolecular Engineering, Korea Advanced Institute of Science and Technology (KAIST), Daehak-ro 291, Yuseong-gu, Daejeon 34141, Korea; nyt0824@kaist.ac.kr (Y.-T.N.); qwerty6063@kaist.ac.kr (J.-D.J.); 2 Department of Chemical and Biomolecular Engineering, Yonsei University, Yonsei-ro 50, Seodaemun-gu, Seoul 03722, Korea; sh010818@yonsei.ac.kr (J.-H.K.); qownsgur10@yonsei.ac.kr (J.-H.B.)

**Keywords:** graphene, nanopore, organic solvent nanofiltration, separation, membrane

## Abstract

Graphene-based membranes are promising candidates for efficient organic solvent nanofiltration (OSN) processes because of their unique structural characteristics, such as mechanical/chemical stability and precise molecular sieving. Recently, to improve organic solvent permeance and selectivity, nanopores have been fabricated on graphene planes via chemical and physical methods. The nanopores serve as an additional channel for facilitating ultrafast solvent permeation while filtering organic molecules by size exclusion. This review summarizes the recent developments in nanoporous graphene (NG)-based membranes for OSN applications. The membranes are categorized depending on the membrane structure: single-layer NG, multilayer NG, and graphene-based composite membranes hybridized with other porous materials. Techniques for nanopore generation on graphene, as well as the challenges faced and the perspectives required for the commercialization of NG membranes, are also discussed.

## 1. Introduction

The organic solvent nanofiltration (OSN) technique has attracted significant attention in separation processes in the chemical and pharmaceutical industries because it can serve as an integrated system, has a high energy efficiency, and exhibits minimal degradation at mild operating conditions [1,2]. In contrast to gas and water separation membranes, OSN membranes must be stable in harsh organic solvents with precise selectivity for small molecules in the nm or Å scales. Thus, graphitic materials such as graphene, graphene oxide (GO), reduced graphene oxide (rGO), and graphene oxide nanoribbons (GONRs) have been used for membrane fabrication because of their excellent chemical stability and mechanical properties, owing to the strong sp^2^ hybridization of carbon atoms [3,4,5,6]. The one-atom-thick, two-dimensional (2D) shape of graphene with a high aspect ratio enables the deposition of an ultrathin selective layer on a porous support, resulting in a high solvent flux. Large-scale fabrication of graphene membranes can be done using continuous coating methods, such as slot-die coating and bar-coating [7,8,9,10]. In addition, molecular separation can be achieved by either a narrow interlayer spacing or nanopores on the basal plane.

Because of the aforementioned reasons, conceptual research on using graphene-based membranes for water treatment has been widely conducted, and a similar approach has been applied to OSN processes [11,12,13]. However, graphene layers are intrinsic barriers that also prevent the permeation of hydrogen and helium; therefore, the low permeation of organic solvents has been reported without any structural modification [14,15]. Thus, numerous approaches have been attempted to enhance the OSN performance by modifying the graphene structure. The application areas of the OSN process have been thoroughly discussed in previous studies; therefore, this review focuses on the current techniques used for tailoring the pore structure of the graphene layer and its effect on the OSN performance. Future perspectives of NG and current challenges faced are also discussed.

**Figure 1:**
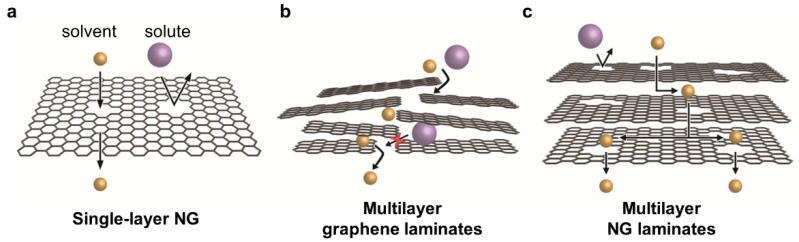
**Three categories of graphene-based membrane used for OSN:** (**a**) single-layer NG, (**b**) multilayer graphene laminates, and (**c**) multilayer NG laminates.

Recently, several graphene-based membranes that are used for water treatment or gas separation have been reported [16,17,18,19,20]. While single-layer nanoporous graphene (NG) membranes have been reported for their significantly thin selective layers, GO-based membranes have been reported, owing to their easy fabrication and precise molecular separation by narrow interlayer spacing. Compared to the research on water treatment membranes, the research on graphene-based OSN membranes is at an early stage. When only graphene is used for membrane fabrication, three approaches can be classified depending on their membrane structure: single-layer NG, multilayer graphene laminates, and multilayer NG laminates (Figure 1).

## 2. Methods for Introducing Nanopores in Graphene

Intrinsically, pristine single-layer graphene is a barrier that prevents the permeation of small molecules, including helium (He), hydrogen (H_2_), and water. This is ascribed to the high electron density of its aromatic rings in the basal plane [14,15,21]. In addition, when GO is reduced to rGO, the effective interlayer spacing of rGO is negligible, and, thus, the small gas molecules do not penetrate the barrier [22]. The interlayer spacing of GO can be tuned by changing the type of organic solvent and the degree of swelling (Figure 1b); however, the molecules of the organic solvent are larger than those of water, and, thus, the permeation resistance to organic solvents can be much higher than to water. Therefore, a low or barrier-like organic solvent permeance was reported using a GO-based membrane, which can be beneficial for the dehydration of organic solvents [23,24].

**Figure 2:**
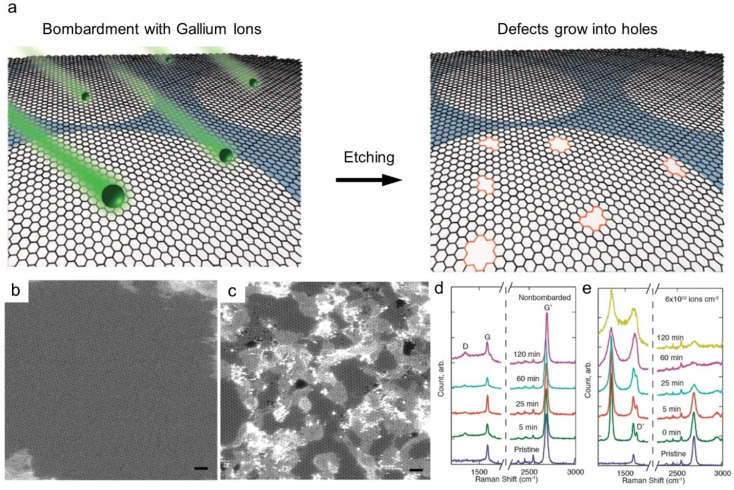
**Membrane made of CVD-grown single layer graphene** (**a**) Nanopore generation by ion bombardment followed by chemical oxidation. (**b**,**c**) TEM image of single layer graphene (**b**) and after (**c**) ion bombardment. Scale bars are 1 nm. (**d**,**e**) Raman spectra depending on chemical etching time and ion bombardment [25]. Copyright 2014 American Chemistry Society.

Nanopore generation is a direct and effective method for enhancing the solvent permeance of graphene-based membranes. For single-layer graphene prepared by chemical vapor deposition (CVD) (Figure 1a), various top-down methods, such as focused ion beam, focused electron beam, ultraviolet-induced oxidative etching, ion bombardment followed by chemical etching, and oxygen plasma etching, have been used [25,26,27,28,29]. After transferring the CVD-grown graphene layer to a porous support, nanopores can be introduced by physical or chemical etching. For example, accelerated gallium ions generate defects on the plane, and further strong etching enlarges the pores (Figure 2a). As shown in Figure 2b,c, the formation of randomly distributed nanopores on the graphene layer can be confirmed by scanning transmission electron microscopy (STEM). By varying the exposure time in the chamber, the pore size and density of the nanopores can be tuned, as confirmed by the increased intensities of the D and D’ peaks in the Raman spectra (Figure 2d,e). Because single-layer or few-layer graphene is used as the selective layer, the membrane thickness can be significantly decreased to the atomic scale. However, as such processes are performed under high-vacuum conditions and post-treated in a chamber, most studies are limited to a small-scale fabrication, while Yang et al. demonstrated its fabrication up to cm-scale [30].

As an alternative method, the nanopores can be generated in GO on a large scale by post-treatment of GO powder; some examples of post-treatments are MnO_2_ etching, KOH activation, H_2_O_2_ treatment, and rapid thermal treatment [31,32,33,34,35]. Intrinsic defects and holes are present in the original GO, formed during the synthesis of GO, but the size and density of the pores are negligible and not controlled. Because such post-treatments generate nanopores by the decomposition of oxygen-functional groups or defective sp^2^ carbon on the GO plane, dense sp^3^ carbon are formed near the pores, and oxygen-functional groups are also partially present. The defective structure is critical to tune the interlayer spacing to enable the permeation of large organic solvents through the nanochannel. Moreover, because NG is soluble in water and organic solvents, it is beneficial to fabricate laminated membranes by solution processes such as vacuum filtration, spin coating, bar coating, spray coating, and slot-die coating (Figure 1c). As observed by transmission electron microscopy (TEM), the size of most nanopores generated by post-treatment was in the range of several nm, while the sizes of the sub-nm-sized micropores can be determined by gas adsorption. The pore size distribution of NG after post-treatment is typically broad; thus, the development of a more precise pore control method is required for the fabrication of highly selective membranes. On the other hand, the bottom-up synthesis of NG has also been attempted by surface-assisted aryl-aryl coupling reactions. However, membranes cannot be fabricated using synthesized graphene because the sheet is insufficient for deposition on a porous support. Thus, the achieved quality of exfoliation of graphene is unsatisfactory [36].

## 3. OSN Performance of NG-Based Membranes

The latest 2D material-based membranes and their OSN performance, including solvent permeation, solute rejection, and molecular weight cutoff (MWCO), are summarized in Table 1. The material types are categorized into transition metal carbides and nitrides (MXenes), transition metal dichalcogenides (TMDs), covalent organic frameworks (COFs), metal organic frameworks (MOFs), layered double hydroxides (LDHs), boron nitride, and NG-based membranes. All these membranes included a common thin-film composite-type membrane in which a selective graphene layer was fabricated on a porous support. Because the graphene layer is typically thin, on the nm scale, an additional freestanding porous support is essFiguretial for ensuring the mechanical stability of the membranes. Although the size and density of the porous support can affect the OSN performance, we focused on the material type of the selective layer, considering the optimized performance under each condition. As the viscosity of the solvent is inversely proportional to the permeance, the filtered solvents are also listed in the table. The details of the NG-based membrane are discussed below.

### 3.1. Single-Layer NG

Single-layer NG membranes have been proposed as ideal membrane models by molecular simulation [62,63]. Their ultrafast transport phenomena were observed to be several orders of magnitude higher than those of the existing polymeric membranes. The well-defined nanopores can act as a precise molecular sieve by size exclusion, demonstrating exceptional rejection, including that of monovalent ions. Pore size directly affects the separation performance. Pore chemistry also significantly affects the separation performance, owing to the electrostatic interaction between the pores and target molecules. For instance, hydroxylated nanopores enable faster water transport than hydrogenated pores, owing to the attraction of hydrogen bonding at pore edges. On the other hand, the ion rejection of hydroxylated nanopores is less than that of hydrogenated pores because of the lower free-energy barrier. O’Hern et al. reported the selective ion transport in water using a single-layer NG membrane fabricated by the ion bombardment of gallium ions, followed by chemical etching [25]. Cracks and pinholes were generated on freestanding single-layer graphene by accelerated gallium ions; then, the defects were grown by oxidative etching. The nanopores with diameters of 0.4 nm and densities exceeding 10^12^ cm^−2^ were randomly distributed on basal planes. The separation performance was investigated using an osmotically driven process at different concentrations of the osmotic solution. The prepared membrane showed cation-selective transport, owing to the electrostatic repulsion between negatively charged pores. Moreover, the large nanopores allowed the transport of salt but rejected the larger organic dye molecules, indicating steric size exclusion. Surwade et al. also introduced nanopores on single-layer graphene using oxygen plasma etching and confirmed the selective ion permeation for salt water desalination [26]. Moreover, the nanofiltration performance under pressures up to 50 bar was examined by Qin et al. [64]. The intrinsic defects were sealed with polymers, and the permeable layer showed an ultrafast water flux of 500 L m^−2^ h^−1^ at 10 bar. In short, they confirmed the feasibility of using single-layer NG membranes in liquid media.

**Figure 3:**
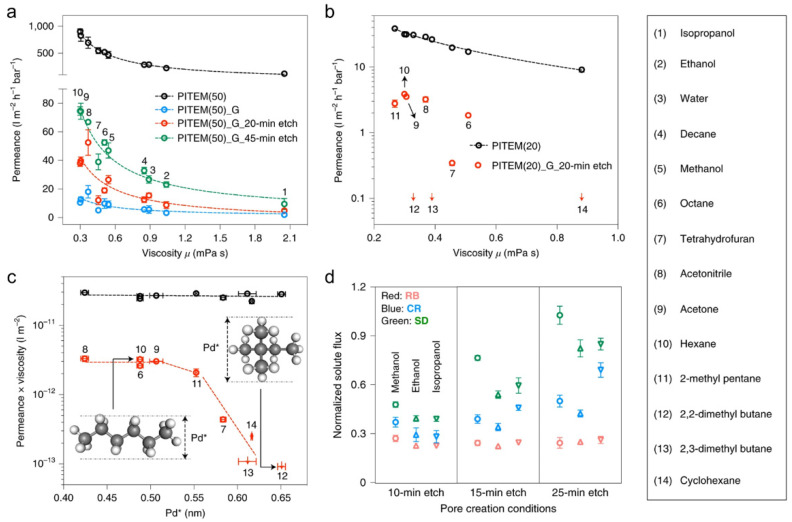
(**a**,**b**) Permeance–viscosity plot for nanoporous graphene membranes fabricated with various pore creation conditions and supported by PITEM (50 nm pore). (**a**) and PITEM (20 nm pore). (**b**,**c**) Dependence of the product of viscosity and permeance on the smallest permeable solvent diameter, Pd*, for PITEM (20)-supported membrane. (**d**) Selective solute diffusion for different-sized dye molecules depending on pore size [50]. Copyright 2021 Nature Publishing Group.

While simulation and experimental results based on the water system are not directly related to OSN, a high organic solvent permanence is expected, while porous support with strong chemical stability is required for further application in OSN, which is another limitation of using single-layer graphene membranes in OSN processes. Cheng et al. demonstrated the use of single-layer NG as an OSN membrane and systematically analyzed continuum flow in the pores [50]. The nanopores were generated using ion irradiation and chemical etching on CVD-grown monolayer graphene, which was then interfacially polymerized to block intrinsic defects. The prepared membrane roughly followed a viscosity-controlled continuum flow, as shown in Figure 3a. In addition, the overall permeance increased with increasing etching time, owing to the formation of larger nanopores. However, some solvents deviated from the plot around the isomer solvents (Figure 3b,c). In particular, the permeation of hexane through the graphene membrane was 3.88 L m^−2^ h^−1^ bar^−1^ and less than 0.1 L m^−2^ h^−1^ bar^−1^ for its branched isomer, 2,2-dimethyl butane. This can be explained by the introduction of Pd*, which is the smallest dimension of molecules: the linear-shaped isomer (hexane) with small Pd* molecules can pass through the pores, but the round isomer (2,2-dimetyl butane) with large Pd* molecules cannot. Therefore, although the viscosities of the two isomer solvents are similar, the permeances × viscosity plot can be different, depending on the geometry of the solvent molecule. The as-prepared membrane demonstrated selective solute diffusion at different etching times, as shown in Figure 3d. Even the 25 min-etched sample was not permeable to large RB molecules, but the flux of small Sudan I (SD) increased with increasing pore size. The practical OSN performance confirmed that a selectivity of over 20 for SD over RB was maintained under pressure-driven filtration in an RB/SD mixed solution in ethanol. Furthermore, stable permeance of approximately 30 L m^−2^ h^−1^ bar^−1^ was exhibited until 381 h.

Although such studies have realized an ideal model using single-layer NG, the solvent permeance was still lower than the theoretical value. This is attributed to the solvent pathway being hindered by the dead area of the porous support with low porosity. A robust support layer is imperative for single-layer membranes to withstand high pressure, but rational design is also required for enhancing permeance. Several studies have shown that the surface properties of the support, such as surface morphology and hydrophilicity, can influence the OSN performance. For example, the water permeance of ultrathin GO membranes (~20 nm) on a wrinkled support was increased by a factor of 6.4, owing to the free volume between the selective and the support layers [65]. In addition, the surface-functionalized GO membrane exhibited a separation factor that was six times larger than pristine GO membrane for the water/butanol solution in the pervaporation system [66]. Thus, other factors, as well as pore structure, should be precisely controlled, along with the development of large-scale fabrication methods. Meanwhile, as CVD-grown graphene inevitably forms grain boundaries, uniform single crystalline graphene seems to be favorable for improving the OSN performance [67].

### 3.2. Multilayer Graphene Laminates

Compared to single-layer NG membranes, multilayer graphene membranes are considered to be more feasible because most fabrication processes are based on solution processes. As a constituent, GO nanosheets are easily soluble in aqueous media and/or some organic solvents; this enables the use of various solution-based fabrication processes, such as vacuum filtration, bar coating, spray coating, spin coating, and slot die coating [10,58,68,69,70]. Utilizing the structural properties of the 2D shape of the GO nanosheet, the laminated structure can be readily aligned by exerting external forces, such as pressure, shear force, and centrifugal force. The resulting interlayer spacing between the laminates can sieve undesired molecules by size exclusion while allowing the penetration of molecules smaller than the interlayer spacing. Thus, various manipulations, from the chemistry of building blocks to fabrication methodologies, have been researched to control the size and uniformity of 2D channels [71,72,73].

Huang et al. demonstrated the semipermeability of multilayer graphene laminates by modulating the interlayer spacing and oxidation degree by thermal annealing [74]. Depending on the polarity of the solvents, structural changes were observed, owing to the different solvation effects. Furthermore, Zheng et al. systematically analyzed the relationship between interlayer spacing and separation capability by predicting the Hansen solubility distance between GO and various solvents [75]. While solvents with small solubility distances (e.g., dimethylformamide (DMF), N-methyl-2-pyrrolidone (NMP)) caused a significant increase of up to 2.7 nm of the interlayer spacing, solvents with a large solubility distance did not affect the interlayer spacing. Notably, the solvents slip significantly faster with an increase in the solubility distance while maintaining a high rejection rate for organic dyes. This indicates that the MWCO of the GO membrane can be significantly modulated, depending on the type of organic solvent. Additionally, the GO layer may be unstable and delaminated without adequate structural modifications, such as the crosslinking of the adjacent GO nanosheets. Interestingly, even after the expansion of the interlayer spacing of GO sheets by solvent swelling, the organic solvent permeance in water was not as high as expected, which may be attributed to the uneven surface of the GO layer comprised of smooth sp^2^ carbon domains and rough oxidized sp^3^ carbon domains, in addition to the interaction between the organic molecules and hydrophobic regions of GO. This observation indicates the importance of GO structure modification for achieving high-performance GO membranes with high flux and precise molecular separation.

**Figure 4:**
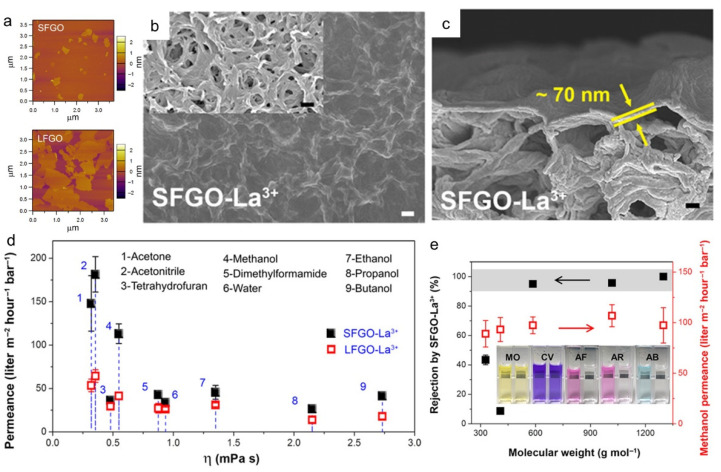
**Graphene membrane comprised of small flake GO (SFGO).** (**a**) Atomic force microscopy (AFM) topographies showing the height images of the small-flake GO and large-flake GO (LFGO) nanosheets. (**b**,**c**) Field-emission scanning electron microscopy (FESEM) images of the (**b**) surface and (**c**) cross-sectional morphologies of the SFGO-La^3+^ membrane (white scale bars, 1 μm; black scale bars, 200 nm). Inset of (**b**): FESEM image of the underlying nylon substrates (black scale bars, 1 μm). (**d**) Permeance of pure water and various pure organic solvents through the SFGO-La^3+^ and LFGO-La^3+^ membranes as a function of their viscosities. (**e**) Separation performances of the SFGO-La^3+^ membrane using various 10 ppm solutions containing organic dyes of different charges and molecular weights of methanol [76]. Copyright 2020 The American Association for the Advancement of Science.

Nie et al. investigated the effect of the flake size of GO on the OSN performance and used La^3+^ cross-linked SFGO to enhance stability [76]. As shown in Figure 4a, SFGO with a lateral size of ~200 nm was prepared by ultrasonication and centrifugation using LFGO. An ultrathin SFGO-La^3+^ membrane with a thickness of 70 nm was prepared on a porous nylon support (Figure 4b,c). The as-prepared membrane showed higher permeance than the LFGO-La^3+^ membrane for all tested solvents because the SFGO-laminated membrane provided short tortuous pathways leading to a faster transport of solvent molecules (Figure 4d). In addition, a sharp MWCO of approximately 586 g mol^−1^ was achieved (Figure 4e). However, the achieved solvent permeance was lower than the expected value for the thin graphene membrane. Although SFGO can provide an enlarged interlayer spacing and short permeation channels, the interlayer spacing remains too narrow for bulky organic molecules, and the presence of oxygen-functional groups may hinder the permeation of the organic molecules.

### 3.3. Multilayer NG Laminates

Cohen-Tanugi et al. prepared multilayer NG membranes using molecular dynamic simulation. [77]. The effects of pore size, pore density, and interlayer spacing on molecular transport were systematically analyzed. In contrast to single-layer nanoporous membranes, interlayer spacing, and pore alignment can significantly affect the permeance of multilayer nanoporous membranes. Based on calculations, an increase in pore size results in high permeation, but this enhancement can also be negligible at narrow interlayer spacings and misaligned pores. This implies that the solvent passing through the plane can be blocked by an excessively narrow 2D nanochannel; therefore, the overall permeance is reduced. When this structure was expanded to a 200 nm-thick multilayer NG membrane, the permeability was approximately 2 L m^−2^ h^−1^ bar^−1^, which is comparable to that of a commercial polyamide membrane. Thus, a comprehensive design of the structure, including pore size, pore density, and interlayer spacing, is required to optimize the function of nanopores as an additional pathway.

Kim et al. synthesized functionalized multilayer NG by treating partially oxidized graphene powder with KOH at temperatures above 600 °C and further oxidizing it to enhance water dispersibility [31]. The pore size ranged from 2 to 4 nm, with an average size of 3 nm; this was directly observed using high-resolution transmission electron microscopy. Water transport was effectively enhanced by the abundant nanopores serving as additional water pathways, while sub-nm-sized dye molecules were rejected by the narrow interlayer spacing (~9 Å). In this work, nanopores only acted as a water pathway and not as a molecular sieve because of the large pore size. This work also demonstrates that introducing additional nanopores is effective for enhancing the solvent permeance of multilayer graphene membranes.

**Figure 5:**
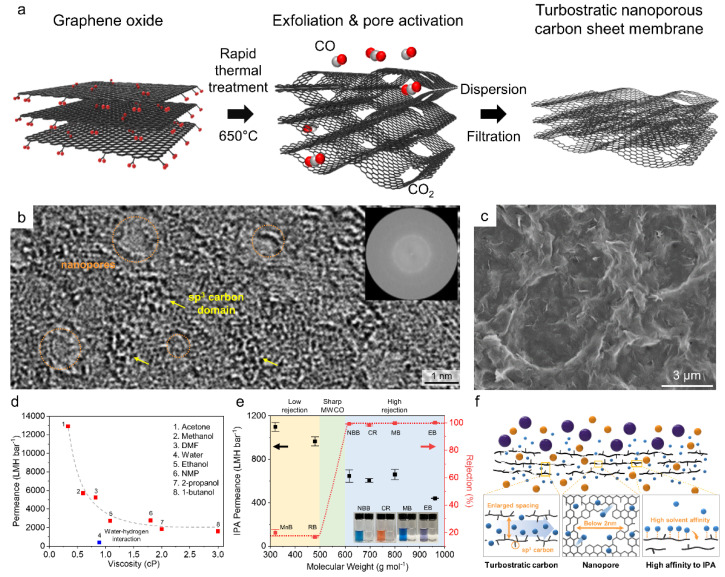
**Thermally activated NG for OSN.** (**a**) Schematic of the rapid thermal treatment of GO nanosheets for pore generation. (**b**) TEM image of the basal plane of the activated GO and the corresponding FFT image. (**c**) Scanning electron microscopy (SEM) image of surface of the multilayer NG membrane. (**d**) Pure solvent permeance as a function of solvent viscosity. (**e**) Rejection rate of isopropyl alcohol (IPA). (**f**) Schematic of the molecular separation mechanism of the multilayer NG membrane [32]. Copyright 2020 Royal Society of Chemistry.

**Figure 6:**
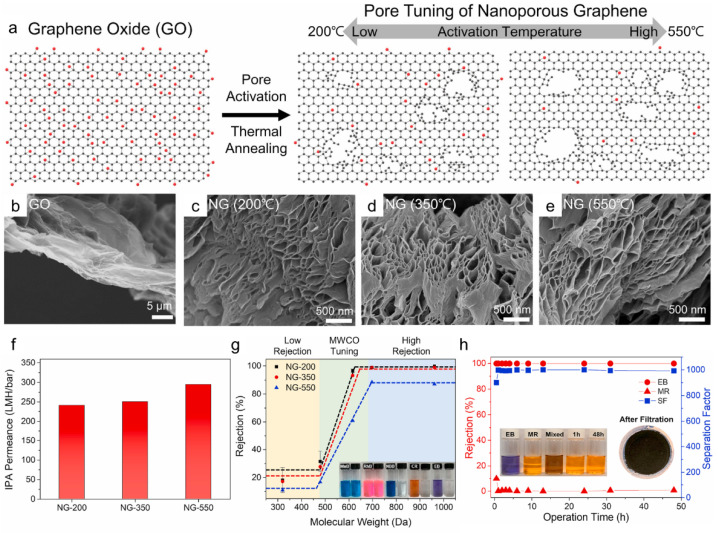
(**a**) Schematic illustration of the pore tuning of NG. (**b**–**e**) SEM images of the GO, NG-200, NG-350, and NG-550 particles, respectively. (**f**) Pure IPA permeance of the NG membranes. (**g**) IPA nanofiltration performance of NG membranes tested with dye molecules, including MnB, RhB, NBB, CR, and EB at 3 bar. (**h**) Long-term filtration performance of the NG-200 membrane under cross-flow filtration for 2 d [61]. Copyright 2021 Elsevier.

As an alternative method, Jang et al. demonstrated the preparation of NG nanosheets by the rapid thermal treatment of GO powder at 650 °C [32]. The oxygen-functional groups present on the basal planes of GO decomposed to CO and CO_2_, forming nanopores on the basal plane of graphene (Figure 5a). When the activated graphene was exfoliated and dispersed in DMF or NMP, a multilayer NG membrane could have been fabricated by filtering the nanosheet solution using a commercial nylon filter. As shown in the TEM image (Figure 5b) of GO, nanopores several nm in size are distinctly observed on the basal plane, which can be detected by Ar adsorption isotherms. Because the activated graphene is mainly composed of amorphous sp^3^ carbon, the stacked layer shows a turbostratic nanochannel similar to those in molecular carbon sieve materials made by the pyrolysis of the polymer (Figure 5c). The fabricated membranes showed ultrafast solvent permeances for most organic solvents inversely proportionate to viscosity (Figure 5d). In particular, 1800 L m^−2^ h^−1^ bar^−1^ of IPA permeance was achieved with a sharp separation curve ranging from 500 to 600 Da, which is significantly greater than that of the existing membranes and single-layer NG membrane (Figure 5e). This excellent permeation is attributed to the widened interlayer spacing by turbostratic carbon, additional solvent pathways by dense nanopores, and relaxed free energy by high solvent affinity to IPA (Figure 5f). Following the first demonstration of membrane fabrication using activated graphene, Kang et al. achieved pore tuning of NG by adjusting the activation temperature (Figure 6a) [61]. Because the decomposition of oxygen-functional groups is significant, and the etching of graphene edges is facilitated at higher temperatures, larger nanopores were observed above 550 °C, while mostly micropores were observed in activated graphene at 250 °C (Figure 6b–e). When membranes with identical selective layer thicknesses of approximately 750 nm were prepared, the pure IPA permeance increased from 241 to 295 L m^−2^ h^−1^ bar^−1^ as the activation temperature increased, owing to larger and denser nanopores (Figure 6f), while smaller MWCO values (lower than 600 Da) were achieved by the low-temperature treatment (Figure 6f). Notably, the main rejection mechanism of the NG membranes seems to be size exclusion by the size of nanopores rather than interlayer spacing, as in GO laminates. Moreover, steady IPA filtration performance was achieved in a cross-flow system for 2 d, demonstrating that the use of multilayer NG membranes may be feasible for separation processes, such as solvent exchange, chemical purification, and solvent purification, in the chemical, semiconductor, electronic, and pharmaceutical industries (Figure 6h).

### 3.4. Graphene Nanoribbon Membrane (Nanopore Made of Entangled GN Nanoribbons)

**Figure 7:**
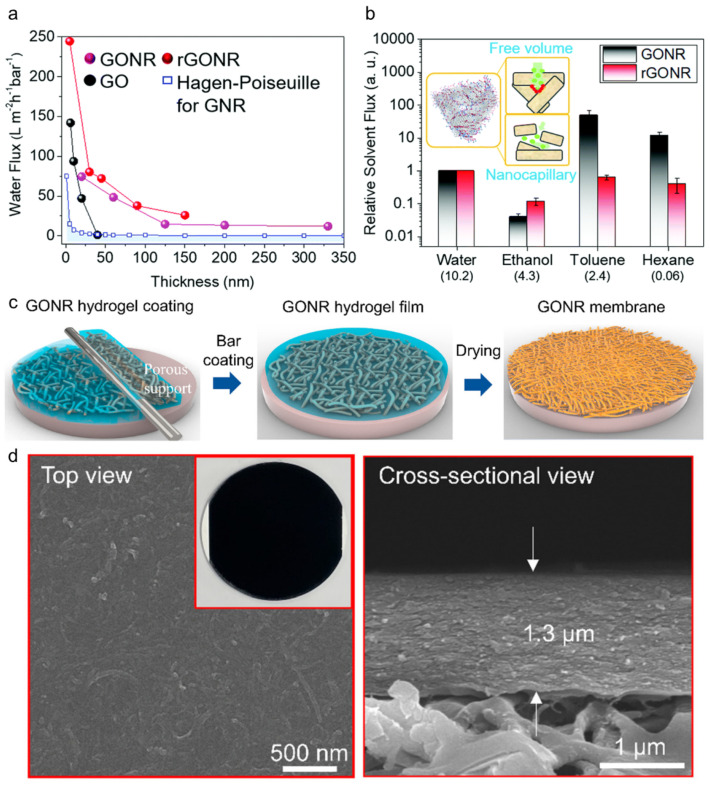
(**a**) Flux of pure water through the graphitic carbon membranes (GONR, rGONR, and GO) at different membrane thicknesses. (**b**) Relative flux of various solvents through the GONR and rGONR membranes of 100 nm thickness, normalized with water flux [78]. Copyright 2017 Royal Society of Chemistry. (**c**) Schematic of the GONR layer-coating procedure. (**d**) Top/cross-sectional SEM images of the GONR film after drying [8]. Copyright 2020 American Chemical Society.

Similar to GO nanosheets, GO nanoribbons (GONRs) are one-dimensional graphitic carbon nanosheets that can be fabricated by the oxidation of carbon nanotubes (CNTs). Kim et al. first suggested the use of laminated GONR membranes for molecular separation in water and various organic solvents [78]. Regardless of membrane thickness, this membrane, prepared by simple vacuum filtration, exhibited a significantly higher water flux than the normal GO membrane, owing to the short diffusion pathway through the laminates and free volume between the entangled nanoribbons (Figure 7a). In addition to structural advantages, the abundance of oxygen-functional groups at numerous edge sites owing to the high-aspect ratio affects solvation, resulting in a preferred diffusion pathway depending on polarity (Figure 7b). For example, polar solvents, including water and ethanol, prefer to flow through the nanocapillary channels rather than the free volume. On the other hand, nonpolar solvents not related to hydrogen bonding are more likely to flow through the free volume between the stacks. In addition, stable rejection of sub nm-sized dyes (100%) and trivalent ions (greater than 60%) was achieved. In this study, significant mechanical and chemical stabilities under harsh conditions, such as acidic, basic, and high pressure, were also demonstrated.

For a scalable fabrication method, Choi et al. analyzed the viscoelastic behavior of GONR hydrogels and developed a facile coating method utilizing this property (Figure 7c) [8]. As the concentration of the GONRs increased, the viscosity of the GONR suspension increased. Finally, the GONR suspension appeared to be a hydrogel at a concentration of 50 mg mL^−1^. This phenomenon is ascribed to the strong hydrogen bonding between the abundant oxygen-functional groups of the GONRs. These characteristics were utilized to uniformly coat a porous support with the GONR films using a bar or doctor blade, and the thickness was controlled by adjusting the concentration of the GONR hydrogel. Finally, a three-dimensional macroporous GONR aerogel with a thickness of 1.3 μm was obtained by freeze-drying (Figure 7d). Finally, the as-prepared GONR membrane exhibited moderate nanofiltration performance with an MWCO of less than 269 Da and was operated steadily under a high pressure and a practical cross-flow system. Because such studies confirmed the superior stability of the GONR membranes in harsh organic solvents, it can be further developed for its application in the OSN field.

### 3.5. Graphene-Based Hybrid Membranes

**Figure 8:**
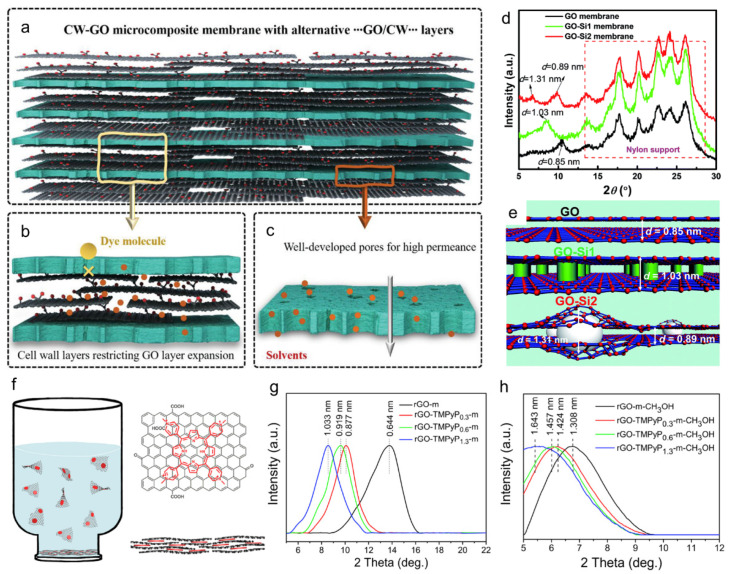
**Graphene-based hybrid membranes** (**a**–**c**) Schematics of the CW-GO microcomposite membrane structure and the working principle of the CW layer between GO layers and CW layer itself [53]. Copyright 2021 Wiley. (**d**) XRD patterns and (**e**) schematic of the GO-Si hybrid membranes, showing the different interlayer spacings [54]. Copyright 2019 Royal Society of Chemistry. XRD patterns of the rGO-m and rGO-TMPyPn-m membranes at (**f**) dry state and (**g**) solvated state in methanol. (**h**) Schematic of the fabrication of an rGO-TMPyPn-membrane with molecularly modulated nanochannels [56]. Copyright 2017 Elsevier.

Because the permeations of organic solvents are low, owing to the tight 2D nanochannels and uneven graphene surfaces, precise control of the interlayer structure is required. More importantly, the interlayer structure of multilayer graphene membrane readily exhibits swelling due to solvation, and, thus, the performance degradation is inevitable in the long-term operation. To control the interlayer spacing, as a prevalent strategy, hybrid membranes mixed with other functional materials have been widely studied. Generally, intercalated molecules expand the interlayer spacing, and electrostatically charged molecules contract the nanochannel by attracting the layers. Zhang et al. fabricated an alternative cell wall (CW)/GO composite membrane to restrict the swelling of the GO layer using the strong interaction between the CW and GO (Figure 8a–c) [53]. Moreover, the in-plane pores of the CW facilitated solvent transport through the plane, resulting in a high permeance of 58.1 L m^−2^ h^−1^ bar^−1^ and a high dye rejection rate of >96% for 120 h. Wang et al. fabricated a dual-spacing channel membrane intercalated with sub-5 nm-silica nanoparticles in GO interlayers [54]. Two distinct peaks of 0.89 and 1.31 nm were confirmed by X-ray diffraction (XRD) patterns (Figure 8d), indicating that the GO-Si2 membrane possessed dual 2D nanochannels, as depicted in Figure 8e. Large nanochannels effectively enhanced solvent permeance, while narrow nanochannels precisely rejected dye molecules and exhibited a permeance of 290 L m^−2^ h^−1^ bar^−1^ and a rejection of >90% for RB and CR. Similar to Si nanoparticles, the hollow structure of ZnS polyhedral crystals after the sulfurization of ZIF-8 generated free volume between the GO layers and successfully enhanced permeance, as reported by Wang et al. [79].

When focusing on stability rather than permeance or rejection rate, molecular interactions between GO nanosheets, such as π-π interactions on the plane and electrostatic interactions at the edge, are further utilized to connect the GO nanosheets to each other using functional materials. Gao et al. crosslinked a boronic acid polymer with GO to form dialkyl phenyl boronate linkages and enhanced stability in organic solvents [55]. The optimized membrane maintained a high rejection of >95% for organic dyes (EB, AF) after being soaked in water for a month. Mahalingam et al. incorporated diamine into the GO layers to fix the interlayer structure through amide bonding and demonstrated a stable OSN operation for 14 h [80]. In addition to diamine, polyethyleneimine, which is a typical polymer with abundant amine groups, was used to connect the GO layers to prevent swelling [81]. Gao et al. intercalated porphyrin molecules, which can self-assemble, into GO nanosheets via π–π interactions (Figure 8f) [56]. Although the fabricated hybrid membrane exhibited swelling in the methanol solution (Figure 8g,h), a steady OSN performance was achieved for up to 25 h. Along with cross-linking between the GO layers, the normal polymer support was replaced with porous freestanding CNT films to enhance permeation as well as mechanical stability, as reported by Kim et al. [82].

## 4. Conclusions and Prospects

In summary, this review elaborated the state-of-the-art techniques for constructing NG-based membranes and their applications in the OSN process. Although research on single-layer NG membranes has been conducted on a limited micro-scale, well-defined structures can be a great platform for understanding the transport phenomena through the pores, providing further insights into the design of pore structures. In the section on multilayer graphene laminate membranes, various approaches to control the interlayer spacing were investigated with respect to OSN performance and stability. The multilayer laminate membrane assembled using NG resulted in significantly fast solvent transport via additional solvent pathways.

Despite the significant improvement of OSN performance in NG-based membranes, several key issues remain to be resolved, considering the existing polymer-based OSN membranes. In terms of pore engineering, the fabrication of homogeneous nanopores is required to achieve a sharp MWCO. Moreover, owing to the wide range of sizes of products used in chemical and pharmaceutical engineering, precise control of pore size is imperative for the design of stepwise separation processes depending on the desired molecule, such as the active pharmaceutical ingredient. In addition, the cost efficiency should be considered for scalable fabrication.

For the optimization of nanoporous structures, practical investigations must be conducted to realize their industrial applications. Excellent mechanical and chemical stability for long-term operation is required for the polymer support layer as well as the selective layer. Because polymer support typically degrades in harsh organic solvents, applying other solvent-resistant support layers is a possible approach. To precisely examine the feasibility of the actual OSN operating in cross-flow systems at high concentrations, membrane evaluation in a mixed-solute system for various organic solvents used for synthesis is essential. Large-scale fabrication using continuous techniques, such as slot-die, bar, and spray coating, should be developed with reliable module assembly. Finally, the optimization of the operating conditions is required to prevent performance degradation, such as concentration polarization and membrane fouling on the surface in a practical cross-flow system. In short, the above challenges must be thoroughly addressed to establish commercialization at the industrial level, and we anticipate that this review will provide insights to accomplish that in the near future.

## Figures and Tables

**Table 1:** Summary of the organic solvent nanofiltration performance of 2D material-based membranes.

Type	Material	Permeance ^(a)^	Rejection ^(^^b)^	MWCO ^(^^c)^	Fabrication Method	Filtered Solvent	REF
MXene	Ti_3_C_2_Tx	982.7	AY79 (100%)	992	Vacuum filtration	Isopropanol	[37]
	PAN/PEI-Ti_3_C_2_T_x_-NH_2_	3	PEG800 (96%)	200	Drop casting	Isopropanol	[38]
	Ti_3_C_2_T_x_/CNTs-CTAB	60.8	CR (>95%)	697	Vacuum filtration	Ethanol	[39]
TMD	S-MoS_2_	636	MnB (99%) MB (100%)	320	Vacuum filtration	Isopropanol	[40]
	D-MoS_2_	109	MnB (98%) BF (100%)	320	Vacuum filtration	Isopropanol	[40]
	WS_2_-NMP-15	44.4	EB (99%) RBB (91.5%)	627	Pressure-assisted filtration	Ethanol	[41]
	(3PEI/5PSS-2.5MoS_2_)_1.5_	3.4	MnB (93.4%)	320	Layer by layer	Ethanol	[42]
COF	TAPA-TFP	127.3	BBR (94.8%)	826	Interfacial reaction	Ethanol	[43]
	GO/COF	50.8	MnB (99%) CR (99.8%)	320	Vacuum filtration	Ethanol	[44]
	SWCNT/COF	60.5	BBG (93%)	854	In-situ growth	Acetone	[45]
MOF	Zn-TCPP (Fe)	140	BBG (90%)	854	Vacuum filtration	Isopropanol	[46]
LDH	Mg-AlLDH	651	AF (99.6%) MO (98.3%)	327	Vacuum filtration	Acetone	[47]
	NiS_2_/Ni-AlLDH	2464	MO (99.9%)	327	Vacuum filtration	Acetone	[48]
BN	FBN-2	330 †	CR (>99%)	697	Vacuum filtration	Ethanol	[49]
	FBN-8	240 †	MnB (93%)	320	Vacuum filtration	Methanol	[49]
Single-layer NG	Defect-sealed NG	170.7	RB (97.3%)	826	CVD	Methanol	[50]
		50.9	RB (95.9%)	826	CVD	Ethanol	[50]
Multilayer graphene	TPP/GO/HPEI	8.5	AB (95%)	1299	Pressure-assisted filtration	Ethanol	[51]
	PA/cGO/cross-linked PI	4.9	EY (100%) RhB (99.3%)	479	Immersion	Ethanol	[52]
	CW-GO	58.1	EB (96%)	248	Vacuum filtration	Methanol	[53]
	GO-Si2	290	RB (91.9%) CR (95.8%)	697	Vacuum filtration	Methanol	[54]
	GO-0.5BA-T	4	AF (95.8%)	586	Vacuum filtration	Methanol	[55]
	rGO-TMPyP_0.6_-44	5.3	EB (>99.9%) AF (92.2%)	586	Vacuum filtration	Methanol	[56]
	GO/EDA	0.5	VB (95.3%)	837	Pressure-assisted filtration	Isopropanol	[57]
	Shear-aligned GO	130	RhB (91%)	974	Gravure printing	Isopropanol	[58]
	Micro & nanosized GO	109.5	MnB (98%) FB (91%)	338	Vacuumfiltration	Isopropanol	[59]
	PI/GO	107.8	OII (96.3%) RB (99.9%)	350	Pressure-assisted filtration	Acetonitrile	[60]
Multilayer NG	TNCS	1839	NBB (99%)	600	Vacuum filtration	Isopropanol	[32]
	NG-200	241	EB (99.9%) CR (98.9%)	616	Vacuum filtration	Isopropanol	[61]

AY79, Acid yellow 79; CR, Congo red; PEG, polyethylene glycol; MnB, methylene blue; MB, methyl blue; BF, basic fuchsin; EB, Evans blue; RBB, Remazol brilliant blue R; BBR, Brilliant blue R; BBG, Brilliant blue G; AF, Acid fuchsin; MO, Methyl orange; AB, Alcian blue 8GX; EY, eosin yellow; RhB, Rhodamine B; VB, Vitamin B12; FB, Fuchsin basic; OII, Orange II; NBB, naphtol blue black. PAN, polyacrylonitrile; PEI, polyethyleneimine; CTAB, Cetrimonium bromide; PSS, poly(sodium 4-styrenesulfonate; TAPA, tris(4-aminophenyl)amine; TFP, 2,4,6-triformylphloroglucinol; TCPP, tetra(4-carboxyphenyl)porphyrin; FBN, functionalized boron nitride; HPEI, hyperbranched polyethyleneimine; PI, polyimide; TNCS, turbostratic nanoporous carbon sheet. (a) Pure organic solvent permeance (L m^−2^ h^−1^ bar^−1^). (b) Representative organic molecules used for the filtration test. (c) MWCO: Molecular weight at 90% rejection. † Dye permeance.
